# Digital ischemia and tobacco

**DOI:** 10.11604/pamj.2017.27.104.12932

**Published:** 2017-06-12

**Authors:** Laura Gallo Padilla, Raquel Ríos Fernández

**Affiliations:** 1 Internal Medicine Service, San Cecilio University Hospital, Granada, Spain; 2 Autoinmune Disease Service, San Cecilio University Hospital, Granada, Spain

**Keywords:** Digital ischemia, vasomotor, buerger, thromboangiitis

## Image in medicine

An active smoker 50-year-old male with precedents of arterial hypertension, consulted for a month history of pain and severe vasomotor disorder in the right hand. Physical exam revealed preserved radial pulse and severe cyanosis in all fingers, especially evolved in the fourth and fifth ones (A). Autoimmunity and hypercoagulability studies were requested and normal. An arteriogram of the right arm showed a corkscrew morphology of radial and ulnar arteries, without a complete refill of the palmar arches and with collateral circulation, typical findings of thromboangiitis obliterans (TAO), also known as Buerger's disease. In spite of smoking cessation and an initial improvement with intravenous iloprost and sildenafil infusions, the injuries kept on deteriorating. A thoracic sympathectomy was perform, which allowed to reduce the doses of opiate for pain control, but did not manage to avoid the amputation. TAO is a rare vascular, inflammatory and occlusive disease that affects the small to medium sized arteries, the distal veins and nerves of extremities. The etiology still remains unclear and it affects typically young smoking men. The only therapy that has demonstrated a constant benefit is smoking cessation. Other therapies frequently used are anticoagulants, vasodilators, antiplatelets and/or anti-inflammatories, although in most cases with a more palliative than curative purpose. Prognosis is favourable if patient quits smoking for good and the vessels damage is not too much evolved.

**Figure 1:**
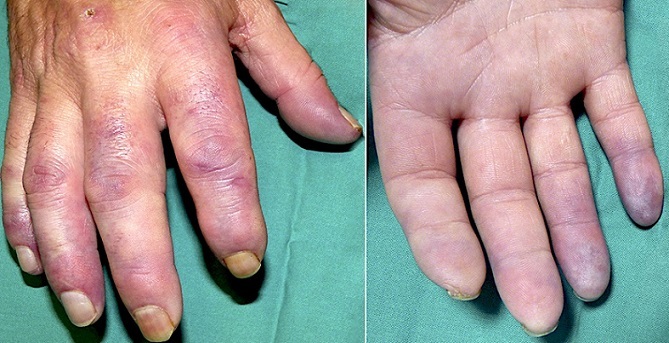
Vasomotor disorder showing severe cyanosis affecting all fingers

